# Transcriptomic Profiling Highlights Metabolic and Biosynthetic Pathways Involved in In Vitro Flowering in *Anoectochilus roxburghii* (Wall.) Lindl.

**DOI:** 10.3390/genes16020132

**Published:** 2025-01-24

**Authors:** Shuisheng Yu, Julian Liu, Chenchen Cai, Yi Zhang, Shuangbin Fu, Yanping Yang, Zhuang Zhou, Zhen Ying

**Affiliations:** 1 Zhejiang Jiulongshan National Nature Reserve Management Center, Lishui 323312, China; schhc@sina.com (S.Y.);; 2 Zhejiang Institute of Subtropical Crops, Wenzhou 325005, China; 3 Innovation Center of Chinese Medical Crops, Zhejiang Academy of Agricultural Sciences, Hangzhou 310021, China

**Keywords:** flower, RNA-seq, metabolic pathways, biosynthetic pathways, DNA replication

## Abstract

Background: Tissue culture is one of the most important methods for propagating orchids. Notably, many orchid seedlings exhibit autonomous flowering during the cultivation process. To explore the underlying mechanism, *Anoectochilus roxburghii* (Wall.) Lindl., an orchid that spontaneously forms in vitro flowers, was analyzed in this study. Methods: Bud samples at the early, middle, and fully open stages were collected for transcriptome sequencing, followed by differential expression, trend, enrichment and protein–protein interaction (PPI) network analyses. Results: Differential gene expression analysis identified 2364, 4137, and 6522 differentially expressed genes (DEGs) in the early vs. middle, middle vs. open, and early vs. open comparisons, respectively. These DEGs were significantly enriched in various metabolic and biosynthetic pathways, particularly in ko01100 (metabolic pathways). PPI network analysis further identified hub genes, including *MCM3*, *MCM4*, and *MCM7*, which are associated with DNA replication, and *CURL3*, which is linked to plant hormone signal transduction pathways. Conclusion: Our findings provide novel insights into the molecular mechanisms driving in vitro flowering in *A. roxburghii*, highlighting the importance of metabolic and biosynthetic process signaling in this unique developmental transition. These results provide valuable resources for future studies on orchid propagation and floral development.

## 1. Introduction

*Anoectochilus roxburghii* (Wall.) Lind. (*A. roxburghii*), commonly known as the Jewel orchid or Jin Xian Lian in Chinese, is a valuable medicinal plant in the Orchidaceae family. Its value lies in its pharmacological properties and increasing market demand [1]; however, this is contrasted by its vulnerability (https://cites.org/eng/disc/text.php, accessed on 12 January 2025) and limited production, making its conservation and cultivation a pressing issue. Research has demonstrated that *A. roxburghii* is a rich source of diverse bioactive compounds, including polysaccharides, flavonoids, amino acids, mineral elements, polyphenols, alkaloids, saponins, and volatile oils [2,3]. In particular, kinsenoside, a pharmacologically active compound found in *A. roxburghii*, has been shown to possess the ability to repair damaged islet cells and restore normal insulin secretion [4].

Tissue culture is one of the most important methods for propagating orchids such as *A. roxburghii*. Previous studies have demonstrated that regenerable seedlings can be obtained through direct organogenesis [5] or indirect (callus) organogenesis [6]. For example, shoot bud induction was achieved on half-strength Murashige and Skoog (MS) [7] medium supplemented with 1.5 mg/L 6-benzyladenine (BA) using nodal segments [5]. Following a similar approach, we successfully regenerated plantlets by culturing nodal segments derived from aseptically germinated seedlings on MS medium supplemented with 20 g/L banana pulp, 30 g/L sucrose, and 5 g/L agar. Notably, a substantial proportion of these regenerated plantlets exhibited autonomous flowering, a phenomenon also known as in vitro flowering, during the in vitro culture period.

In vitro flowering refers to the induction of spontaneous flowering under controlled conditions [8]. Unlike natural flowering, which is influenced by environmental variables and often characterized by long juvenile periods or irregular cycles, in vitro culture allows for the manipulation of factors such as photoperiod, temperature, and hormonal balance. This enables the induction of flowering independent of seasonal variations. Such control is crucial not only for the rapid clonal propagation of elite genotypes but also for facilitating biochemical and genetic studies of the flowering process itself, making it an ideal system for studying the mechanisms behind plant flowering [9]. As a result, many researchers have actively attempted to induce in vitro flowering by adjusting the composition of the culture medium. For example, BA, a synthetic cytokinin (CK), is widely used for the artificial induction of in vitro flowering [10]. In *Dendrobium candidum*, 82.8% of shoots formed floral buds on MS medium supplemented with 0.01 mg/L BA [11]. Similarly, in *Dendrobium nobile*, 1.0 mg/L BA was effective for floral bud induction (20.0%) [12].

In natural environments, phytohormones also play crucial roles in regulating flower initiation and development [13]. For instance, Gibberellins (GAs) promote flowering and stem elongation, often by influencing *FLOWERING LOCUS T* (*FT*) gene expression and DELLA protein degradation [14]. Auxins affect inflorescence architecture and floral organ development, interacting with other hormones like CKs [15]. CKs are essential for flower meristem identity and organ development, often acting antagonistically to auxins [16].

Furthermore, factors such as juvenility, ambient temperature, and photoperiod also significantly influence flowering time in plants [17,18]. Juvenility involves changes in GA signaling, miRNA regulation, and carbohydrate metabolism [19]. Ambient temperature affects flowering through epigenetic modifications during vernalization and influences alternative splicing and hormonal signaling [20]. Photoperiod is sensed by photoreceptors, which interact with the circadian clock to regulate the expression of *CONSTANS* (*CO*) and *FT*, ultimately leading to flowering [21]. These processes are intricately linked to various metabolic pathways, highlighting the complex interplay between metabolism, development, and environmental cues in controlling flowering. In particular, carbohydrate metabolism, phenylalanine-related pathways, and starch, sucrose, and fructose metabolism were significantly enriched. Similar pathways have been identified during the flower development processes of Petunia [22], *Paeonia lactiflora*, [23] and tea [24].

MADS-box genes, encoding a family of transcription factors characterized by the conserved MADS domain, play pivotal roles in plant development, particularly in floral organ identity. Within this family, *FLOWERING LOCUS C* (*FLC*) and *SHORT VEGETATIVE PHASE* (*SVP*) are two well-characterized floral repressors. *FLC* primarily delays flowering by directly repressing the expression of downstream flowering promoters, such as *FT*. In *Arabidopsis thaliana*, vernalization (prolonged exposure to low temperatures) effectively silences *FLC* expression, thus relieving its repressive effect on flowering and promoting the transition to the reproductive phase [25]. *SVP*, also a MADS-box transcription factor, often interacts with *FLC* to form heterodimeric complexes, synergistically repressing the expression of flowering-related genes and contributing to the precise control of flowering time. Studies have shown that *SVP* can also function independently of *FLC*, participating in other regulatory pathways, such as temperature response [26]. The regulation of floral organ identity is elegantly explained by the ABC model, initially established in *Arabidopsis* and *Antirrhinum* [27]. This model proposes a combinatorial action of three classes of genes (A, B, and C) to specify the four basic floral organs: sepals, petals, stamens, and carpels. However, the floral architecture of orchids, characterized by unique structures such as the labellum (a modified petal) and the fused column (a structure formed by the fusion of stamens and pistil), necessitates a modification of this model. In orchids, the ABC model is often expanded to the ABCDE model to account for these unique features. This expanded model highlights the crucial roles of B- and E-class MADS-box genes in specifying these orchid-specific floral organs. Consequently, variations in the expression patterns and interactions of MADS-box genes are thought to contribute to the remarkable floral diversity observed in orchids [28,29,30,31].

While progress has been made in understanding the molecular mechanisms of natural flower development, the process of in vitro flowering, particularly in orchids such as *A. roxburghii*, remains largely unexplored at the molecular level. Existing studies on natural flower formation may not be directly applicable to in vitro conditions, where environmental factors such as photoperiod, temperature, and hormonal balance are tightly controlled. Despite the potential applications of in vitro flowering in germplasm conservation, clonal propagation, and genetic studies, there is a significant gap in the literature regarding the specific genes and pathways that drive in vitro flower formation in orchids.

In this study, we aim to explore the molecular mechanisms underlying in vitro flowering in *A. roxburghii* by analyzing the transcriptomes of buds at early, middle, and fully open stages. By identifying the key genes and pathways involved in in vitro flower development, our research aims to provide insights into how in vitro conditions influence flowering, contributing to a better understanding of orchid development and informing strategies for manipulating flower induction in the orchid or other species.

## 2. Materials and Methods

### 2.1. Materials

**Figure 1:**
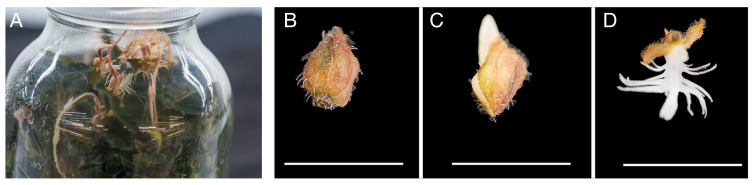
Three distinct developmental stages of in vitro flowers: (**A**) Autonomous flowering of *A. roxburghii*. (**B**) Bud approximately 5 mm long; scale bar = 1 cm. (**C**) Medium-sized bud, approximately 10 mm long. (**D**) Mature, fully open flowers.

*A. roxburghii* seedlings were derived from seeds(Collected from Lishui, China) aseptically sown on MS(Kangbeisi Biology, Hangzhou, China) + banana pulp 100 g/L + activate carbon(Morebetter, Hangzhou, China) 1 g/L + agar(Morebetter, Hangzhou, China) 5 g/L from an artificial hybrid collection(Collected from Lishui, China) which had been cultivated for two years. The nodal explants cut from seedlings were inoculated on MS medium containing 20 g/L banana pulp, 30 g/L sucrose (Morebetter, Hangzhou, China), and 5 g/L agar to obtain in vitro flowering plants (Figure 1A). Bud samples at different stages of development, including the early (early bud, S1) (Figure 1B), middle (medium-sized flower, S2) (Figure 1C), and fully open stages (open flower, S3), were collected (Figure 1D). A minimum of 30 flowers per sample were collected from 10 in vitro-grown seedlings, with 3 samples from each developmental stage. The samples were immediately frozen in liquid nitrogen after harvesting. After storage for 30 min, the flower samples were stored at −80 °C (Ultra-low temperature freezer, Haier, Qingdao, China) for further experimentation.

### 2.2. Library Construction and Transcriptome Sequencing

mRNA was isolated from the samples using mRNA capture beads. After isolation, the mRNA was fragmented using high temperatures. The fragmented mRNA was then used as a template to synthesize the first strand of cDNA in a reverse transcription enzyme mixture system. While the second strand of cDNA was synthesized, end repair and poly (A) tailing were performed. Next, adapters were ligated, and Hieff NGS^®^ DNA Selection Beads were used for purification to select target fragments. PCR library amplification was then performed and, finally, detection was carried out on the Illumina sequencing platform by Genedenovo Biotechnology Co., Ltd. (Guangzhou, China). Each study was performed in three biological replicates.

### 2.3. Differential Gene Expression Analysis

Gene expression levels were expressed as fragments per kilobase of transcript per million fragments mapped (FPKM). Differential expression analysis of groups was performed via DESeq2 (1.42.1) [32]. The resulting *p* values were adjusted via Benjamini and Hochberg’s approach for controlling the false discovery rate (FDR). Genes with an adjusted *p* value < 0.01 and an absolute fold change (FC) ≥ 2 according to DESeq2 in any pairwise comparison (S1 vs. S2, S2 vs. S3, and S1 vs. S3) were considered DEGs.

### 2.4. Annotation

Gene Ontology (GO) annotation was performed via eggNOG-mapper software (2.1.12) and the eggNOG DB [33]. Other functional annotations were performed via the BLASTx program (http://www.ncbi.nlm.nih.gov/BLAST/, accessed on 10 December 2024) with an E value threshold of 1e-5 to the NCBI nonredundant protein (Nr) database (http://www.ncbi.nlm.nih.gov, accessed on 10 December 2024) and the Kyoto Encyclopedia of Genes and Genomes (KEGG) database (http://www.genome.jp/kegg, accessed on 10 December 2024).

### 2.5. PPI Network

The PPI network was constructed via STRING v10 (12.0) [34]. Genes were presented as nodes and their interactions were presented as lines within the network. The network file was further analyzed using the cytoHubba plugin, and the maximum clique centrality (MCC) method [35] was applied to identify the top 10 genes. Finally, the network was visualized via Cytoscape (v3.10.2) [36] software to visualize core and hub gene biological interactions.

### 2.6. Statistical Analyses

The data in this study were analyzed via the R platform (4.4.2) [37]. Data visualizations were generated primarily via the ggplot2 R package (3.5.1) [38]. Principal component analysis (PCA) was performed and visualized via the FactoMineR R package (2.11) [39]. Cluster analysis was performed via the Mfuzz R package (2.62.0) [40], and the results were visualized using the ClusterGVis R package (0.1.1) [41]. Heatmaps were generated via the ComplexHeatmap R package (2.18.0) [42]. Venn diagram was visualized via the ggVennDiagram R package (1.5.2) [43]. The network was calculated via the igraph R package (2.1.1) [44] and visualized using the ggraph R package (2.2.1) [45].

## 3. Results

### 3.1. Gene Expression Analysis

**Figure 2:**
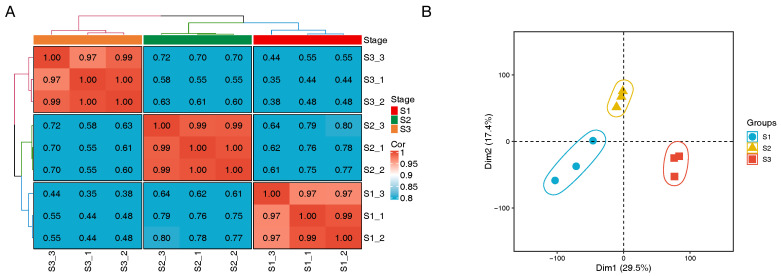
Correlation assessment of biological replicates. (**A**) Correlations between samples. (**B**) PCA of samples.

Pearson’s correlation coefficient was applied in this study to evaluate the reproducibility of the biological replicates, and the results revealed that the correlation coefficients between replicates of each sample were greater than 0.9 and were clustered into three categories: S1, S2, and S3 (Figure 2A). This high degree of correlation indicates strong positive correlation and robust reproducibility within each group. This categorization was further confirmed by subsequent PCA (Figure 2B), which indicated that the three stages of in vitro flowers were clearly distinct.

### 3.2. Differentially Expressed Gene (DEG) Screening

**Figure 3:**
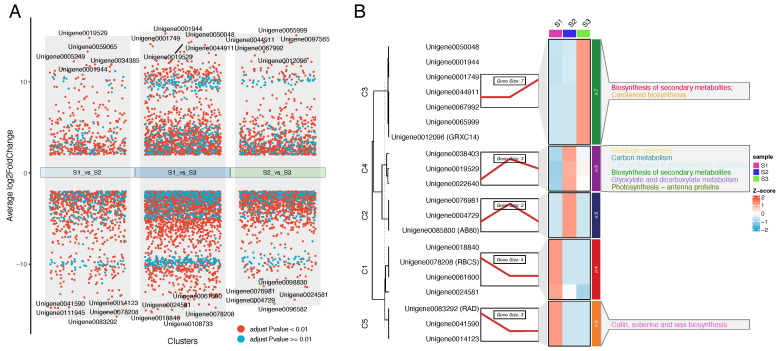
Analysis of DEGs and the top ten DEGs in the three comparisons. (**A**) Volcano plot of the top ten DEGs identified in the three comparisons; the top 20 genes are marked. (**B**) Expression patterns and KEGG enrichment analysis of the top ten DEGs in the three comparisons.

As shown in Figure 3A, all three comparison groups exhibit a large number of DEGs (red points), indicating significant differences in gene expression between the S1, S2, and S3 sample groups. According to the DEG analysis, in the comparison between S1 and S2, 855 DEGs were upregulated and 1509 DEGs were downregulated. In the S1 vs. S3 comparison, 2212 DEGs were upregulated and 4310 DEGs were downregulated. Moreover, in the S2 vs. S3 comparison, 1503 DEGs were upregulated and 2634 DEGs were downregulated. The expression patterns and trends of the top 20 genes revealed that these genes could be clustered into five distinct groups using the R Mfuzz package. KEGG annotation further indicated that metabolic and biosynthesis pathways, such as metabolic pathways and biosynthesis of secondary metabolites, were enriched in many of these genes. Additionally, these genes predominantly clustered in C3 and C4, suggesting that the S2 and S3 groups may be more active in metabolism than the S1 group (Figure 3B).

### 3.3. Trend Analysis

**Figure 4:**
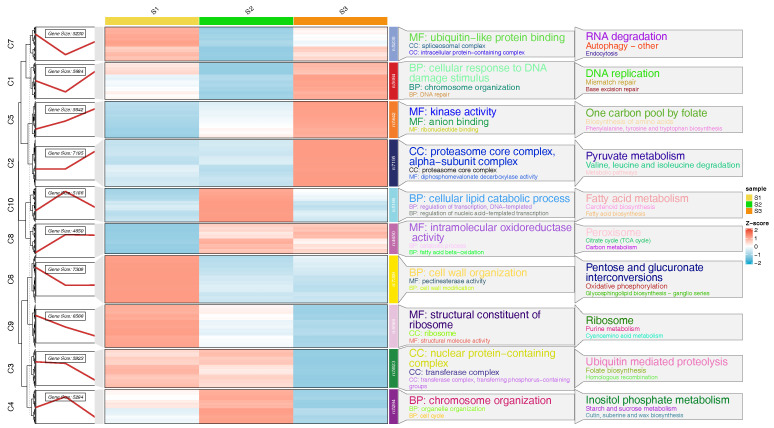
Trend analysis and gene enrichment analysis. The size of the letter represents enrichment significance.

The three time points were artificially clustered into 10 classes, with the number of genes in each cluster and their expression trends illustrated in Figure 4. The S1 group tends to highly express genes in clusters C6, C7, and C9, which are related to functions like ubiquitin-like protein binding, cell wall organization, structural constituent of ribosome, RNA degradation, pentose and glucuronate interconversions, and ribosome metabolism. The S2 group tends to highly express genes in clusters C4 and C8, which are related to functions like cellular lipid catabolic process, chromosome organization, fatty acid metabolism, and inositol phosphate metabolism. The S3 group tends to highly express genes in clusters C1, C2, and C5, which are related to functions like cellular response to DNA damage stimulus, kinase activity, proteasome core complex, alpha-subunit complex, DNA replication, one carbon pool by folate, and pyruvate metabolism. These differences are reflected in the expression levels of gene clusters associated with different functions. For example, the S1 group might be more active in cell wall-related processes, while the S3 group might be more active in DNA replication and protein synthesis.

### 3.4. Enrichment Analysis

**Figure 5:**
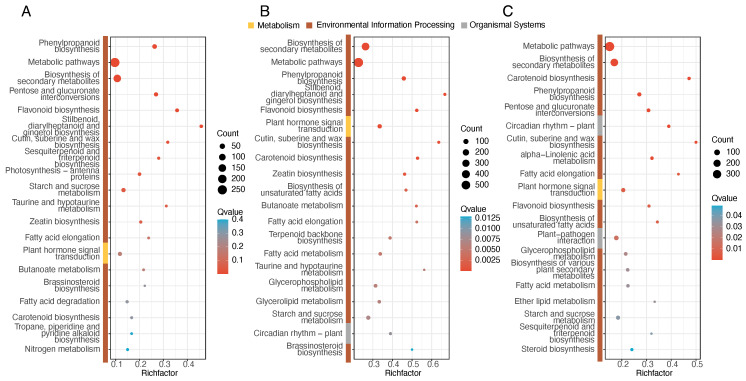
KEGG analysis of three comparisons. (**A**) S1 vs. S2. (**B**) S1 vs. S3. (**C**) S2 vs. S3.

Among all comparisons, the secondary pathways were classified into only three categories: metabolism, environmental information processing, and organismal systems. Regarding the primary classification of pathways, the diagrams show that metabolic and biosynthesis pathways, particularly those related to metabolism and the biosynthesis of secondary metabolites, were significantly enriched with the majority of DEGs across all three comparisons. This suggests that these processes play a central role in the observed biological changes. Additionally, plant hormone signal transduction was enriched in a subset of DEGs across all three comparisons, emphasizing the involvement of hormonal signaling in the in vitro flowering process(Figure 5A–C). The expression and mapping pathways are shown in Figures S1 and S2.

### 3.5. Core Gene Analysis

**Figure 6:**
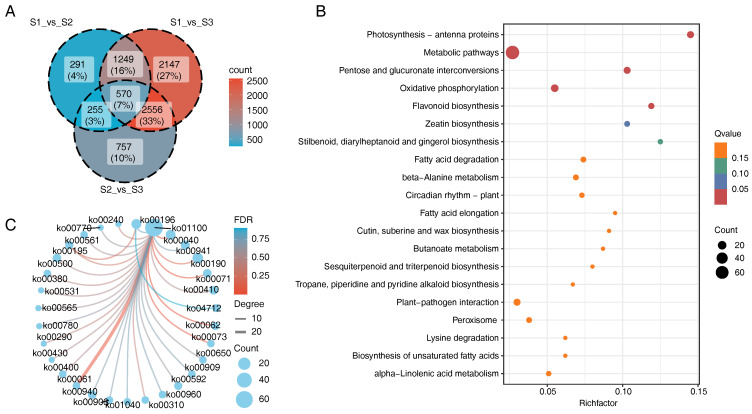
Core gene analysis. (**A**) Venn diagrams of DEGs. (**B**) KEGG pathways of common DEGs. (**C**) Network of KEGG pathways among common DEGs.

According to the KEGG enrichment analysis, the DEGs identified among the different bud samples exhibited significant overlap across the three pairwise comparisons. As illustrated in the Venn diagram (Figure 6A), a total of 570 DEGs were commonly identified in all three comparisons, highlighting their potential shared roles in key biological processes. KEGG pathway enrichment analysis revealed that these overlapping DEGs were predominantly associated with metabolic pathways (Figure 6B).

To further explore the functional relationships of these DEGs, a KEGG pathway network was constructed (Figure 6C). This network analysis demonstrated that most of the DEGs were mapped to the ko01100 (metabolic pathways), a comprehensive pathway encompassing various primary and secondary metabolism processes. Additionally, these genes showed extensive interconnections with other pathways, indicating their involvement in a highly integrated and complex metabolic network. Such connectivity suggests that the identified DEGs may play pivotal roles in coordinating metabolic and biosynthetic activities during in vitro flowering process. For more detailed insights, Figure S3 provides an expanded view of the pathway interactions and connections.

### 3.6. PPI Network

**Figure 7:**
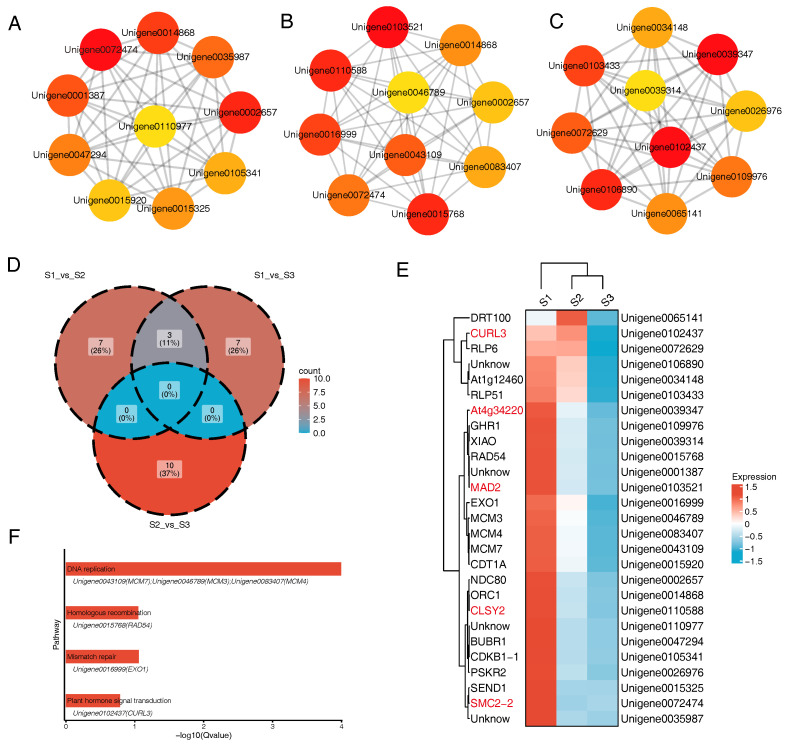
PPI and gene analysis of three samples. (**A**) Top 10 genes and related networks identified via cytoHubba in S1 vs. S2. The shades of color represent the MCC score calculated by cytoHubba. (**B**) Top 10 genes and related networks identified via cytoHubba in S1 vs. S3. (**C**) Top 10 genes and related networks identified via cytoHubba in S2 vs. S3. (**D**) Venn diagram of the top genes in the three samples. (**E**) Expression of the top genes in the three samples. Red indicates hub genes. (**F**) KEGG enrichment analysis of the top genes.

The PPI network derived from the transcriptome data revealed several crucial insights into the underlying biological processes of in vitro flowering. By using cytoHubba, the top 10 genes were identified in each comparison, including Unigene0072474, Unigene0103521, Unigene0110588, Unigene0102437, and Unigene0039347, which were identified as key hub genes with the highest scores within the network (Figure 7A–C). A Venn diagram revealed that there were no common genes among the selected top genes (Figure 7D), indicating that distinct sets of hub genes are active during different stages of in vitro flowering. Further analysis of these top hub genes revealed a general decreasing trend in their expression levels from the early stage to the fully open stage (Figure 7E). Notably, pathway enrichment analysis revealed a significant association of these hub genes with the DNA replication pathway, along with enrichment in other pathways such as homologous recombination, mismatch repair, and plant hormone signal transduction (Figure 7F).

## 4. Discussion

Many researchers have highlighted the critical role of plant growth regulators (PGRs), particularly CKs, in inducing flower formation [9]. However, the tissue culture medium used in our study contained no exogenous PGRs. Therefore, the primary factor influencing in vitro flowering in our system likely resides in the inherent composition of the medium, providing the essential nutrients required for the metabolic processes driving floral development.

Indeed, during the developmental switch from vegetative growth to flowering, plants integrate various signals, including those from light, hormones, and metabolites. Metabolites such as sucrose and starch serve as crucial energy sources and signaling molecules, playing key roles in flower bud formation across multiple flowering pathways [46]. Previous omics studies have revealed dynamic changes in genes, proteins, and metabolites involved in various metabolic pathways in *Dendrobium officinale* [47], apple [48], loquat [49], cabbage [50], and daylily flowers [51]. Similar to these in the natural flowering process, the majority of DEGs were significantly enriched in metabolic and biosynthesis pathways, particularly ko01100 (metabolic pathways) where nearly all common DEGs among the three comparisons were enriched.

In addition to metabolic pathways, the biosynthesis of secondary metabolites was significantly enriched among the DEGs. Previous studies have shown that many secondary metabolites play critical roles in floral induction and the development of individual floral organs during normal growth [52]. Similar pathways have been reported in Petunia, where genes regulating phenylalanine-related pathways such as flavonoid biosynthesis, phenylpropanoid biosynthesis, and phenylalanine metabolism were significantly enriched during the flower developmental stages [22]. Consistent findings in other plant species [53,54] further suggest that secondary metabolites are actively involved in key developmental events at various stages of flower development.

CytoHubba identified the top 10 genes, and hierarchical trend analysis of the top genes across the three samples revealed a broad and gradual decline in expression levels, suggesting that in vitro flowering is a progressive, preparatory process. Further analysis of the five hub genes revealed that *CURL3* (*BRI1*), a brassinosteroid (BR) leucine-rich repeat (LRR) receptor, is involved in signal transduction. Several studies have shown that this gene is associated with flower development and yield. For example, in *Arabidopsis*, the overexpression of a wheat *BRI1* ortholog enhances both flowering and yield [55]. In tomato, *CURL3* is believed to influence floral organ and pollen development by regulating the BR and GA signaling pathways [56]. The KEGG results further support the idea that hormone signaling still plays an important role in flower formation.

Additionally, as shown by the results of KEGG enrichment analysis, three hub genes (*MCM*) were enriched in the DNA replication pathway. *Arabidopsis* and maize are preferentially expressed in young, actively dividing tissues [57,58], reflecting their essential function in cell proliferation. In maize, transgenic studies have demonstrated that MCM6 is crucial for both vegetative and reproductive growth and development [59]. Furthermore, studies in other species, such as *Sinapis alba* [60], *Silene coeli-rosa*, and *Pharbitis nil* [61], have shown that the transition to flowering can be associated with changes in DNA replication dynamics within the shoot meristem, including altered replicon length and origin usage. In the context of our in vitro flowering system, the enrichment of MCM genes likely reflects the high rate of cell division and growth associated with floral bud development. This suggests that, while DNA replication is a necessary supporting process for development, including flowering, it is the metabolic pathways, as discussed previously, that are more likely to be the primary drivers of in vitro floral induction in *A. roxburghii*.

Regarding the phytohormones, in our study, only a small portion of hormone-related genes were identified via the KEGG pathway analysis and the subsequent PPI network analysis. This observation contrasts with some previous research. For example, molecular biology investigations have shown that hormones and their associated genes enriched in hormonal signaling pathways play central roles in the formation of normal and abnormal flowers in vitro [62]. Similarly, in natural environments, transcriptomic profiling of the *Cymbidium sinense* flower development process revealed that gene clusters were also closely associated with these pathways. Additionally, metabolomic analysis revealed 69 potential hormones, with GA and abscisic acid (ABA) acting as key regulatory hubs, whereas GA4 and GA53 exhibited a reciprocal regulatory loop [63]. This complex interplay underscores the intricate hormonal regulation involved in in vitro flowering processes. One possible explain for our findings is that the tissue culture medium used in this study contained no PGRs, meaning the hormonal effects observed likely originated solely from endogenous production in the plants. Additionally, the controlled environment of tissue culture makes the flowering process of *A. roxburghii* distinct from that of other orchids flowering under natural conditions.

Our study presents a preliminary investigation into in vitro flowering. However, there are several limitations to consider. Firstly, our analysis is based solely on transcriptomic data, which provides insights into gene expression levels but not the complete metabolic or hormonal dynamics. Subsequent metabolomic studies and analyses of plant hormone profiles will be crucial for further elucidating the observed transcriptomic changes and offering a more comprehensive understanding of the processes involved in in vitro flowering.

Secondly, our study was conducted under specific in vitro conditions. These conditions, while optimized for in vitro flowering, may not fully reflect the complex environmental cues experienced by plants in their natural habitat. Therefore, the gene expression patterns observed in this study may not entirely reflect those occurring during natural flowering.

Furthermore, limitations in genomic resources and gene transformation technologies for *A. roxburghii* currently hinder the direct functional validation of the identified DEGs through techniques such as gene knockout or overexpression. Future development of these resources will be essential for a deeper understanding of the roles these genes play in the flowering process. Moreover, comparing in vitro flowering with naturally occurring flowering in same plant species is vital to better understand how controlled conditions influence the molecular mechanisms behind flowering.

Despite these limitations, our findings offer valuable insights into potential strategies for improving in vitro flowering in orchids. Based on our preliminary findings, manipulating the hormonal balance in the culture medium could be an effective strategy to improve in vitro flowering rates. Based on our findings, we propose that supplementing the medium with exogenous cytokinins could potentially enhance floral bud development. Furthermore, the observed upregulation of metabolism genes suggests that increasing the sucrose concentration in the culture medium may provide the necessary energy for floral induction. However, further experiments are needed to determine the optimal concentrations and timing of these adjustments for *A. roxburghii* and other orchid species. Future research could also explore the combined effects of hormonal and nutrient adjustments to achieve synergistic effects on in vitro flowering.

## 5. Conclusions

In the present study, we performed transcriptome profiling of in vitro flowering in *A. roxburghii*. Our analysis revealed a significant enrichment of DEGs associated with metabolic and biosynthetic pathways. Combining the top gene-enriched DNA replication pathway suggested a dominant role for plant energy synthesis and metabolism in in vitro flower formation. Our findings provide novel insights into the genetic and molecular mechanisms underlying floral induction and development in this species, identifying both known and previously uncharacterized genes and pathways. These results serve as a valuable resource for future research on both in vitro and natural flowering. However, while our data highlight the importance of energy metabolism, we recognize that it is likely integrated within a complex regulatory network involving other factors such as hormonal signaling and transcriptional regulation. Future research exploring these interactions will be essential to fully elucidate the molecular mechanisms controlling floral transition in *A. roxburghii*.

## Data Availability

The raw RNA-seq data (Accession no. PRJNA1211490) were uploaded to NCBI. Further inquiries can be directed to the corresponding author.

## Supplementary Materials

The following supporting information can be downloaded at: https://www.mdpi.com/article/10.3390/genes16020132/s1, Figure S1. Heatmap; Figure S2. Map of ko04075 (plant hormone signal transduction). A. S1 vs. S2. B. S1 vs. S3. C. S2 vs. S3. Figure S3. Map of ko01100 (metabolic pathways). A. S1 vs. S2. B. S1 vs. S3. C. S2 vs. S3.
