# Supplementary material for: Transcriptomic Profiling Highlights Metabolic and Biosynthetic Pathways Involved in In Vitro Flowering in Anoectochilus roxburghii (Wall.) Lindl
Source: Genes (Basel). 2025 Jan 24;16(2):132. doi: 10.3390/genes16020132 (PMC11855183; doi:10.3390/genes16020132)
Supplement: Supplementary file 1 [file genes-16-00132-s001.zip › Fiure S1 and Figure S2.pdf]

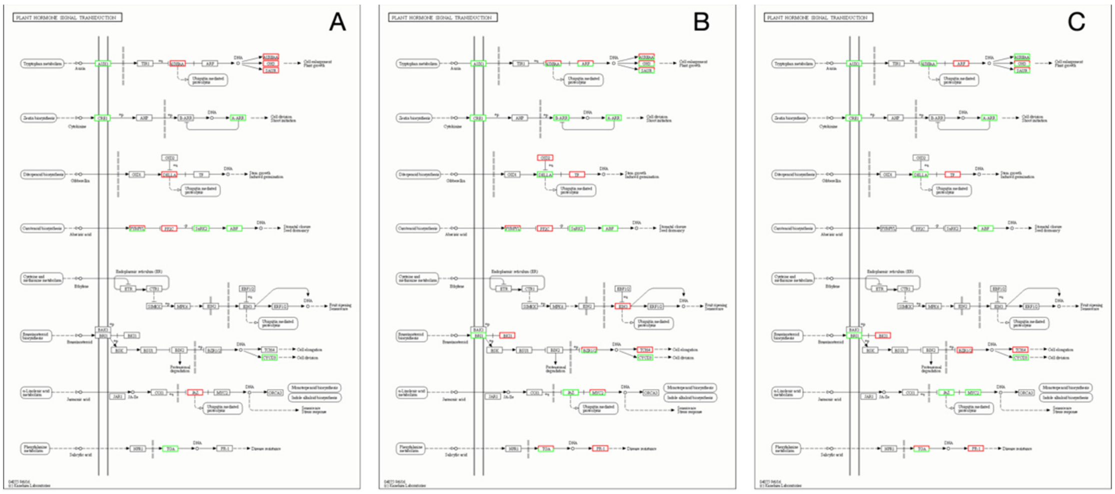

Figure S2. Map of ko04075 (plant hormone signal transduction). A. S1 vs. S2. B. S1 vs S3. C. S2 vs S3.

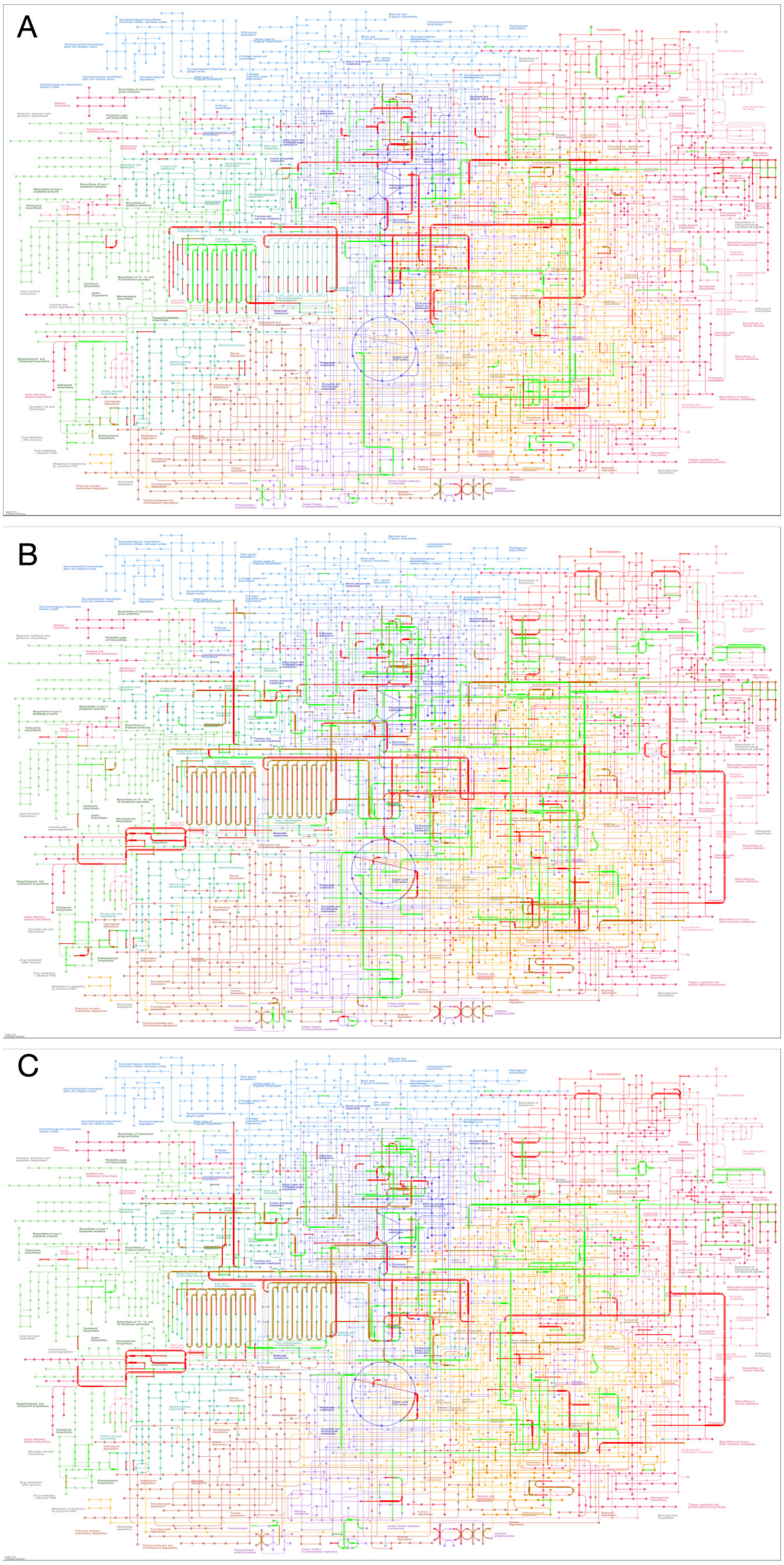

Figure S3. Map of ko01100 (metabolic pathways). A. S1 vs. S2. B. S1 vs S3. C. S2 vs S3.
