# Supplementary figures and images for: Transcriptomic Profiling Highlights Metabolic and Biosynthetic Pathways Involved in In Vitro Flowering in Anoectochilus roxburghii (Wall.) Lindl
Source: Genes (Basel). 2025 Jan 24;16(2):132. doi: 10.3390/genes16020132 (PMC11855183; doi:10.3390/genes16020132)

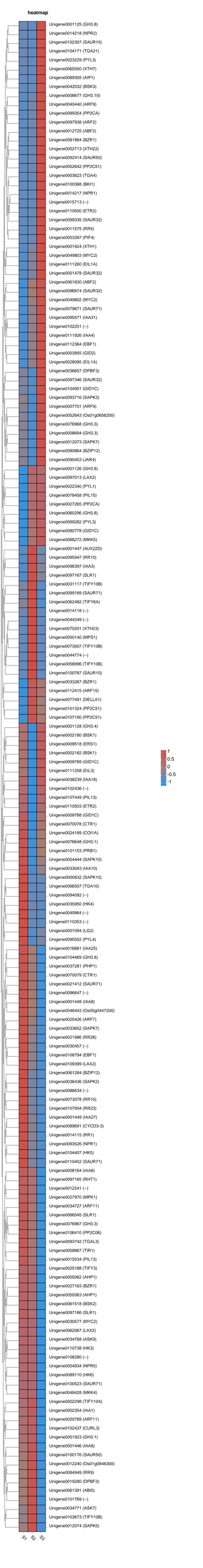

Supplement: Supplementary file 1 [file genes-16-00132-s001.zip › Figure S1 Heatmap.pdf]
